# Correction to “Low-Background Cancer Imaging with a Bioorthogonal Fluorescence Probe and Engineered Reporter Enzyme Bearing a Targeting Moiety”

**DOI:** 10.1021/jacs.6c04784

**Published:** 2026-03-23

**Authors:** Ziyi Wang, Ryosuke Kojima, Rikuki Kiji, Kyohhei Fujita, Ryo Tachibana, Reiko Tsuchiya, Taku Uchiyama, Yoshihiro Minagawa, Tadahaya Mizuno, Kiyohiko Igarashi, Hiroyuki Noji, Mako Kamiya, Yasuteru Urano

In the initially published version, the Michaelis constant (*K*_m_) reported in Figure 3D for Td2F2 WT toward HMRef-β-d-Fuc contained a data entry error. The value was listed as 1.8 × 10^1^ μM, whereas the correct value is 1.9 × 10^2^ μM. In this correction, Figure 3 has been revised to reflect the correct value, while no text in the manuscript has been altered. All the other values in Figure 3D are unchanged. This error was mistakenly introduced during proof revision and reflects an incorrect entry of the *K*_m_ value only; all analyses and discussion in the article, as well as the evaluation during peer review, were performed using the correct *K*_m_ value. Accordingly, the scientific conclusions of the study are entirely unchanged.

**Figure 3:**
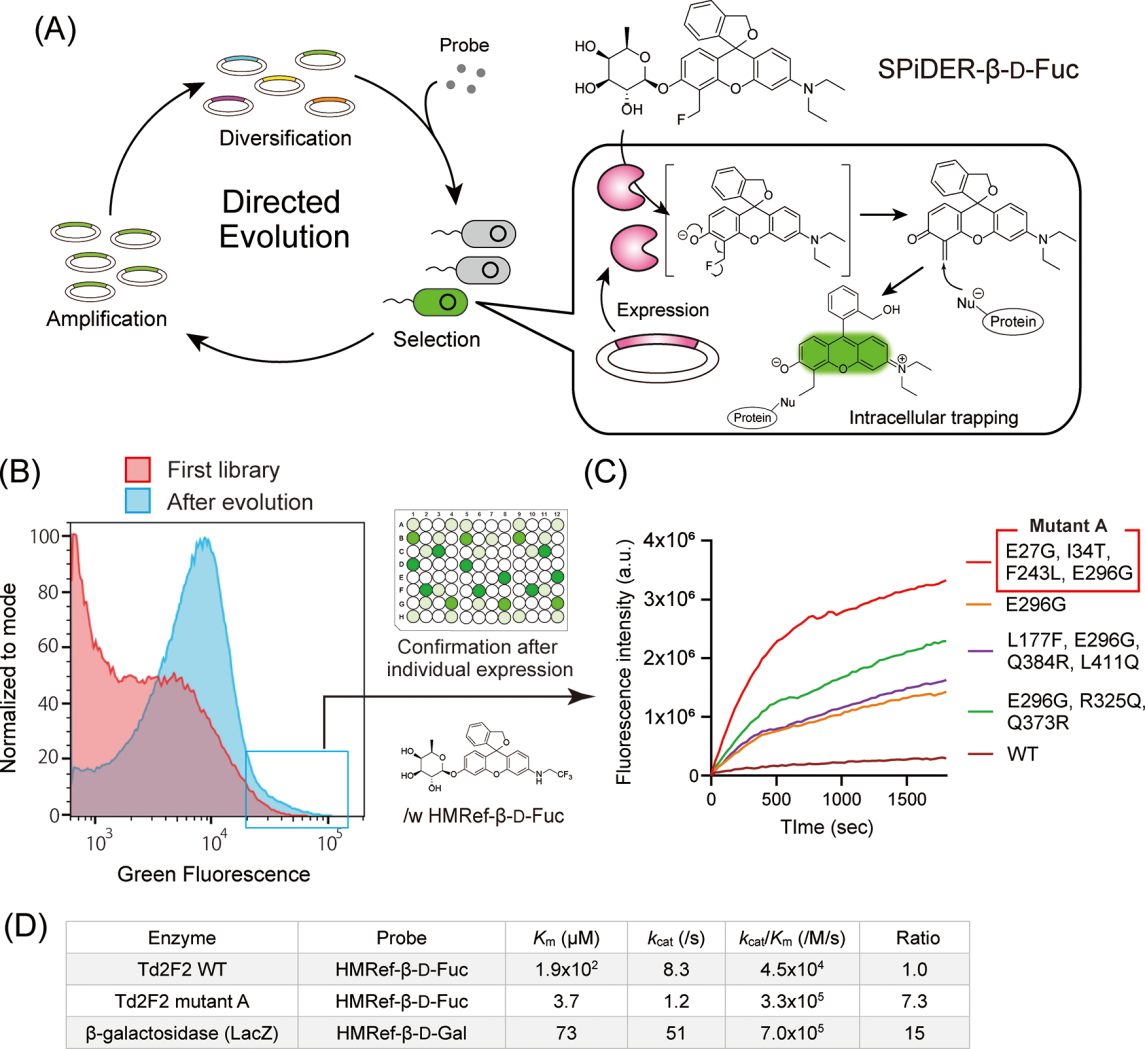
Revised Figure 3.

